# Assessment of the Impact of Salt Iodisation Programmes on Urinary Iodine Concentrations and Goitre Rates: A Systematic Review

**DOI:** 10.1155/2021/9971092

**Published:** 2021-06-02

**Authors:** Almeida Abudo Leite Machamba, Francilene Maria Azevedo, Aline Carare Candido, Mariana de Souza Macedo, Silvia Eloiza Priore, Sylvia do Carmo Castro Franceschini

**Affiliations:** Department of Nutrition and Health, Federal University of Viçosa (UFV), Viçosa/36570.900, Brazil

## Abstract

**Introduction:**

Two main strategies are currently recommended for the prevention and control of iodine deficiency in the world: implementation of universal salt iodisation programmes and permanent monitoring of iodine consumption by the population. Although iodine intake and coverage iodised salt have increased in the world population, iodine deficiency disorders (IDDs) may still be a public health problem in a few countries or communities.

**Objective:**

To assess the impact of salt iodisation programmes on urinary iodine concentrations and goitre rates in the world population. *Methodology.* A systematic review based on the PRISMA method. We obtained articles from Scopus, Science Direct, MEDLINE databases, and other sources between March and April 2020, without limitation of dates. “Iodisation” AND “urinary iodine concentrations” AND “goitre” in English, Portuguese, and Spanish without filters and clinical trial, case-control, and cross-sectional studies were included in this review.

**Results:**

Of 479 abstracts, twenty-three were eligible. Coverage on iodised salt was in the range of 16 to 98%, and 11 studies had been sufficient, whilst eight studies had adequate iodine concentration in salt and three excess. 81.8% of studies that had an adequate median of UIC had a good impact in their respective salt iodisation programmes.

**Conclusion:**

After 18 years of salt iodisation programme implementation in the 13 countries, the majority achieved sustaining elimination of IDD whilst all had adequate median UIC; however, more detailed studies are still needed to confirm that all communities are equally protected of IDD.

## 1. Introduction

Iodine is a micronutrient utilised for the synthesis of thyroid hormones and necessary for neurological development. Iodine deficiency (ID) causes damage to health, such as goitres, hypothyroidism, hyperthyroidism, neuropsychomotor retardation, and cretinism [1]. International organisations suggest two strategies for the prevention and control of ID: implementation of a universal salt iodisation (USI) programme and monitoring iodine consumption by the population through urinary iodine concentration (UIC) assessment. The impact of salt iodisation programmes is assessed by process and impact indicators. Process indicators are associated with the concentration of iodine in salt and how much of the population has access to iodised salt [1]. Impact indicators assess UIC [1], also considering the thyroid volume (Tvol) and the total goitre prevalence rate (TGR). High iodine intake reduces both thyroid volume and TGR, and both of these measures are reported to be more sensitive to long-term iodine intake assessment; hence, they are good indicators [2, 3].

Salt iodisation is a safe, effective, and low-cost strategy, and its implementation is mandatory in 128 of the 196 countries of the world. Of note, the particular programmes vary depending on the conditions in each country [4]. Based on the proportion of cases with a median UIC <100 *μ*g/L, the World Health Organization (WHO) has estimated that 1.9 billion people, corresponding to 31% of the world population, are affected by insufficient iodine intake across 47 countries, where iodine deficiency disorders (IDDs) still remain a health public problem [1]. To achieve sustainable IDD control, global experience has demonstrated that salt iodisation programmes are the most equitable, effective, and sustainable strategy to ensure adequate iodine status for all population groups [3]. It is important to note that the indicator is a median UIC <100 *µ*g/L and not the proportion of cases below that value.

Currently, the global coverage of iodised salt in families is 86%. This has resulted in the number of countries with IDDs being reduced from 113 (1993) to 24 (2017) [3]. Nevertheless, ID may remain in some countries, and the effect of salt iodisation programmes on ID and IDDs in the world population is unclear. Therefore, the objective of this review was to assess the impact of salt iodisation programmes on urinary iodine concentrations and goitre rates in the world population.

## 2. Materials and Methods

This systematic review was based on the Preferred Reporting Items for Systematic Reviews (PRISMA) [5]. The guiding question was what is the impact of salt iodisation programmes on iodine deficiency and iodine deficiency disorder of the worldwide population between 1999 and 2020?

The search occurred in March and April 2020, using MEDLINE (PubMed), Science Direct, and Scopus databases; the Iodine Global Network; the Food Fortification Initiative; the Cochrane library; and the Portuguese Medical Association without limitation of dates. We used the descriptors: “iodisation” and “urinary iodine concentrations” and “goitre”, provided by Health Science Descriptors (DeCS) in English, Portuguese, and Spanish, without filters.

As inclusion criteria, all articles had to have been published between 1999 and 2020, which corresponds to the period during which salt iodisation programmes have been intensely promoted and available in many countries. The studies also had to address the effect of the salt iodisation programmes, detailing the indicator utilised, mean ± standard deviation (SD)/median iodine content in salt, and UIC. Systematic reviews, manuscripts, and articles that assessed iodine content but were not associated with iodised salt consumption and that addressed the iodine nutritional status or TGR of the population prior to the implementation of the salt iodisation programme were excluded.

PICOS was defined as follows: population—people in populations before and after introduction of USI; intervention—USI introduced (iodised salt used, iodised salt coverage, adequate iodised salt used); comparator—historical control period before USI or before intervention; and outcomes—TGR and median UIC after an intervention.

All articles were recorded in a spreadsheet in Microsoft Excel^®^ after completing searches and eliminating duplicates for each database and between databases. Three researchers selected the articles independently. In the case of divergence, the researchers discussed the article and reached an agreement. This review used randomised clinical trials and longitudinal case-control and repeated cross-sectional studies.

The impact of salt iodisation programmes was assessed using the criteria for the elimination of IDDs established by the WHO: (i) the proportion of 8- to 10-year-old children with a median UIC < 100 *μ*g/L, (ii) the TGR of 8- to 10-year-old children is <5%, and (iii) at least 95% of the population has access to iodised salt and >90% of that salt is adequately iodised.

The iodine status was classified as mild for a median UIC < 100 *μ*g/L or adequate for a median UIC between 100 and 199 *μ*g/L. For schoolchildren, the risk of adequate iodine intake was between 200 and 299 *μ*g/L or ≥300 *μ*g/L for risk of adverse health consequences.

Iodine intake in pregnant women was classified as insufficient for a median UIC < 150 *μ*g/L, adequate for a median UIC between 150 and 249 *μ*g/L, above requirements for a median UIC between 250 and 499 *μ*g/L, and excessive for a median UIC ≥ 500 *μ*g/L. For lactating women, a median UIC < 100 and >100 *μ*g/L was considered insufficient and adequate, respectively.

TGR was classified as none if between 0% and 4.9%, mild if between 5% and 19.9%, moderate if between 20% and 29.9%, and severe if ≥ 30% [1, 6, 7].

UIC is a biomarker of iodine status in schoolchildren and adults and an indicator of the iodine intake in schoolchildren, adults, and pregnant and lactating women. Of note, it is also an indicator of the impact of and can represent ID. In addition, TGR represents IDDs and represents thyroid function [8].

The salt iodisation programme was classified as unsuccessful when ID was observed in the population and the median UIC was inadequate. It was classified as inequitable when UIC was adequate but IDDs were present. It was classified as effective when UIC was adequate and IDDs were absent. It was classified as sustainable when the goal of IDD elimination had been achieved [3].

This review followed a method proposed by Downs and Black [9] to assess the quality of the studies. It comprises four categories: report of the study (clarity), external validity (representativeness), internal validity (biases and confounding factors), and statistical power of the study. Only 17 questions of the scale were used because 10 are applicable to experimental studies. The responses score was “1” (when the criterion assessed was present) or “0” (when the criterion assessed was absent).

## 3. Results

**Figure 1:**
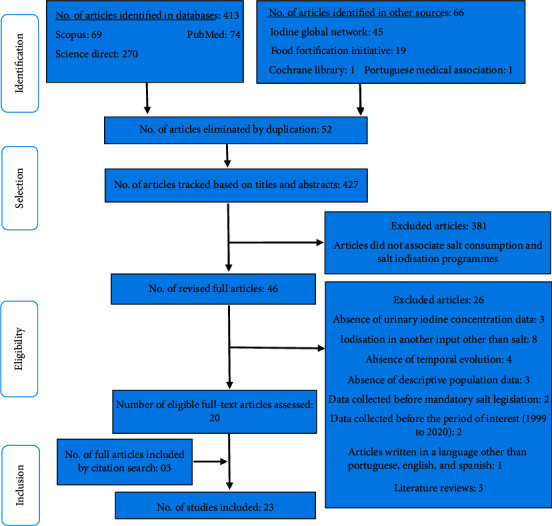
Flowchart showing the process of identification and selection of the included articles.

The search resulted in 479 articles, 403 from the databases and 66 from the other resources. After eliminating duplicates and reading titles, abstracts, and complete articles, we included 20 studies. After a citation search, we included three additional articles, for a total of 23 (Figure 1).

Of the 23 studies assessed, 100% reported changes in the investigated population's median UIC and 68.2% presented changes in TGR (Table 1, continuation I, II, III, IV, and V). The studies provided data from an assessment period from 1999 to 2020 and were conducted in North America, the Eastern Mediterranean region, Africa, South, Southwest and East Asia, and the Western Pacific with infants, school children, and pregnant and lactating women. The sample size varied ranging from 96 to 31 million people. The studies with a low sample size assessed pregnant women and lactating women. The first limitation of studies was low coverage iodised salt in the household of these women and in many cases the women not given sufficient sample salt in collection data. In contrast, in the countries with higher sample sizes, the median UICs were adequate and the TGR decreases.

In studies, the TGR was low in iodised salt consumers (7, 6%) compared with nonconsumers (33%). The UIC was higher in infants with mother consumers of the iodised salt. The UIC was higher in urban people compared to rural, higher in soil iodine concentration areas compared to other areas without, and higher in newborns or teenage girls compared to pregnant women and lactating women, and in pregnancy, the UIC decreased from the first to the third trimester.

Based on the salt iodisation programme classification for schoolchildren, five were sustainable, in China, Iran, Ethiopia, Tanzania, and Turkey [10–21]; five were effective, in Mexico, Tunisia, New Zealand, Portugal, and Sierra Leone [13, 22–24]; and two were inequitable in India and Cameroon [25, 26]. For pregnant women, two were inequitable in Sierra Leone and China [18, 24]. The programme in Italy was the only one without success.

Regarding the assessment of the methodological quality of the studies, the lowest score was 10 and the highest was 17, indicating good quality and reliability of the results. The best-assessed criteria were objectives/hypothesis clearly described, random variability of data for outcomes, sample representativeness, equal follow-up time for the whole sample, valid and reliable outcome measures, individuals recruited in the same population, and individuals recruited within the same time period. However, only five studies described power.

## 4. Discussion

The WHO has defined process indicators for assessing and monitoring fulfilment of salt iodisation programmes as the iodine concentration in salt (ICS) and salt coverage or consumption (SC). The impact indicators to identify the achievement of goals established for the elimination of IDDs are UIC and TGR [1, 34].

### 4.1. ICS and SC

The coverage of iodised salt in the population ranged from 16% to 98%, although in 10 studies, it was sufficient (>90%). However, in the present findings, the ICS varied from 2% to more than 153% of the value recommended by the WHO. Of the 14 studies that assessed ICS, 11 reported an adequate ICS based on the countries' legislation. Of those, eight were within the WHO recommendations (15–40 mg of iodine/kg of salt) and three were in excess. Countries localised in the Eastern Mediterranean and Southwest Asia had less access to iodised salt compared with countries in Africa, North America, and East Asia. However, all the regions were considered to be iodine-deficient areas in 2011 [4]. This review evidenced fulfilment of salt iodisation legislation. Among countries that had reached the goal of iodised salt coverage, there had been a gradual reduction in the iodination range and salt consumption [16, 18, 28, 30].

Regarding outcomes, many studies did not find a difference in the ICS and SC between rural and urban areas. In 2001, in Sparta [27], western Turkey, SC was greater in urban compared with rural areas. However, some regions had excess iodine intake because of high iodine concentrations in drinking water. On the other hand, high iodine intake was observed due to consuming other food sources of iodine such as bread, milk, and dairy products. Consequently, even though people in urban areas showed reduced iodised SC, they still consumed adequate amounts of iodine. One study reported that dairy products and bread contribute 13%–64% of the daily adult iodine intake requirement in high-income countries when consumed more than twice a day per person [35], because, in these countries, a reduction in salt intake has been prioritised to reduce the risk of noncommunicable diseases [36]. These iodine food sources are important as supplements but not as salt substitutes.

Inadequate salt storage and washing salt with water before seasoning foods are some of the practices observed in the home environment that can compromise the amount of iodine available in salt. These are in contrast to the use of crystal salt, which contributes to an adequate ICS. Therefore, it is necessary to maintain the salt iodisation programmes, even with some industries claiming high costs in the production of iodised salt. Governments should provide legislation to ensure that salt iodisation programmes continue and manufacturers comply [37, 38].

### 4.2. UIC

The adoption of universal salt iodisation has been positive for an adequate median UIC in 17 (73.9%) of the 23 studies included in this review [10, 11, 13–26, 30, 31]. Among the six (26.1%) other studies that represent countries with ID, two [27, 28] were assessed in schoolchildren and the other four [29, 31–33] were in pregnant and lactating women; the authors reported insufficient UIC and a moderate level of IDDs.

Based on the studies, a 16.9%–166% increase in UIC (*R*^2^ = 0,143; *P*=0.005) (Supplemental Figure 1) in a population with ID and IDDs within 3–18 years after a salt iodisation programme had been implemented is relevant because it classifies the salt iodisation programmes as promising [39]. In this case, three studies showed good scenarios. A randomised clinical trial showed an 81% increase in UIC levels in iodine-deficient pregnant women and adequate intake 3 years after the salt iodisation programme had been implemented [11]. In the two studies that examined schoolchildren, both started the salt iodisation programmes in 1999. In one, the UIC level had increased 162% 16 years after the implementation and was adequate [14]. On the other hand, the increase was 166%. Five years after implementation (1999–2005), such a level was adequate, but in the subsequent 12 years (2006–2017), it represented an excess [25].

These outcomes also evidence that even with the recommended ICS, ID can still occur in pregnant women. Furthermore, lactating women lose iodine to breast milk to ensure that their newborns have adequate iodine. Hence, the recommended iodine concentration in salt recommended to the overall population [40] is not necessarily sufficient for pregnant and lactating women.

### 4.3. TGR

IDDs were indicated in 15 (65.2%) studies, showing goitre reductions from 1.2% to 62.3% (*R*^2^ = = 0,328; *P*=0.001) (Supplemental Figure 2) between 16 and 18 years after the intervention had commenced. Goitres are still prevalent in the regions where chronic ID has been identified, and this represents an indicator of nutritional status over a long period [34, 41]. Thus, TGR reduction in a population is expected when the intervention is combined with an increased ICS, more of the population has access to iodised salt, and there is regular monitoring of ID in the population. The change in the TGR pattern from severe to mild and from mild to eliminated IDDs in the 18-year period of intervention with iodised salt indicates the adequacy of UIC levels [10, 11, 14–20].

Regions where IDDs had been eliminated showed a reduction in the iodine concentration in salt over time [20]. This phenomenon was observed in the Eastern Mediterranean region, which was previously classified as an area with ID, and, curiously, sub-Saharan Africa in 1999, when the USI programme was recommended by the WHO. Recent studies have reported excess UIC and have recommended readjusting the ICS [42, 43]. This phenomenon may be present in populations with ID and sufficiency iodine intake. The consumption of iodised salt is crucial for the prevention and control of IDDs. Countries should examine their iodine policies to ensure that iodine-deficient areas have greater access to iodised salt.

The data confirmed that pregnant women represent a unique group when assessing UIC in the adult population. Specifically, IDDs occur more in women than in men, and they are 10 times more likely to occur in young than old women, especially in pregnant women living in areas with insufficient iodine levels [44, 45]. However, the occurrence of IDDs in women is associated with impaired brain development in their children [6].

The outcomes showed adequate UIC in schoolchildren, but goitres were present in areas with iodine deficiency as well as adequacy, with a higher incidence in those groups that did not consume iodised salt. Hence, monitoring the nutritional status and nutritional intake of the population is crucial [1].

Some regions of the countries with IDDs in the population presented higher iodine content in the water because these people previously had iodine deficiency [46].

### 4.4. Classification of Salt Iodisation Programmes

The studies that reported adequate UIC underscored the good impact that the salt iodisation programmes had had. Indeed, three programmes [13, 21, 25, 26, 31] were inequitable, six [22–24, 30] were effective, and nine [10, 11, 14–20] were sustainable, thus reducing or eliminating IDDs [1, 6, 34]. Most studies examined schoolchildren, who are particularly vulnerable to iodine insufficiency and sufficiency [3]. The salt iodisation programmes have had a positive impact on that population [10, 11, 13–26, 30]. Two studies also showed a positive impact in pregnant women [18, 24]. However, the salt iodisation programmes in four countries had not shown an impact on pregnant [29, 31–33] and lactating women [31]. According to the United Nations Children's Fund (UNICEF), reaching an adequate iodine content in salt is one of the major steps to prevent and control IDDs. Indeed, this achievement indicates that there are robust policies, including legislation requiring effective monitoring of iodisation, communication, strong industry partnerships, and freedom of a country to manipulate iodine content based on salt iodisation legislation [39].

### 4.5. Salt Iodisation Programmes in Different Regions

The recent recommendations from UNICEF for salt iodisation programmes differ within the same country (rural and urban areas) across time (number and time of the monitoring programme) and depending on the iodine concentration in salt or soil, the iodine deficient population (children or women of reproductive age), socioeconomic status, and regions (south, north, east, and west) [3].

#### 4.5.1. Eastern Mediterranean, South, Southwest Asia, and Africa

In Iran, salt iodisation programmes became sustainable for schoolchildren 11 years after implementation, based on the increased iodised salt coverage. After two years in northwest Iran with 20–40 mg iodine/kg salt, there was ID in rural areas but excessive iodine in urban areas; however, in the northern part of the country, salt iodisation programmes were achieved sustainable in 2015. In the southern part of the country, there was insufficient UIC in pregnant and lactating women, although newborns were iodine adequate. The iodisation programmes were classified as sustainable because the SC was increasing even though the ICS was 63% of the recommended value, a discrepancy that underlies why ID is still observed in the country.

Other findings showed achievements a short time after the intervention had been introduced. In Ethiopia, the salt iodisation programme was sustainable after 3 years, based on the increased iodised SC and ICS. Since 2010, the ICS has been readjusted. Indeed, since 2013, the ICS has been set at > 15 mg iodine/kg salt for rural areas in the northern part of the country, and the salt iodisation programme has been sustained [33]. Ethiopian children whose mothers consumed iodised salt had adequate iodine concentration [11]. In 2014, some villages in the north had been exposed to 20–40 mg iodine/kg salt, and this was inadequate: ID and IDDs appeared in pregnant women. However, the country has achieved sustainable elimination of IDDs in the population. In a 2018 study from China, there had been sustainable elimination of IDDs through salt iodisation in children [20]. Studies from 2017 in Aira, in rural areas in western Ethiopia, showed 34% and 80% of mild ID in 73 schoolchildren and 40 pregnant women surveyed [47]. In this case, the salt iodisation programme could not be sustained in this country. Whilst this study was not representative of the country, it provides directions for the need to monitor the iodisation programme over time.

In India, the salt iodisation programme, with 20–40 mg of iodine/kg salt, was inequitable 1 year after it began for adult women in the north [26]. However, pregnant women in the centre of the country with >15 mg iodine/kg salt had ID, although there had been greater salt coverage and more than half the adequate ICS. This was the only case where pregnant women had an elevated risk of ID still with adequate ICS. This risk is likely due to the low ICS in rural regions and low consumption of iodised salt [27, 29, 30]. The ICS for this group has been readjusted in some countries, including China, because this group requires more iodine compared to the rest of the population [48].

#### 4.5.2. East Asia and Europe

In the four studies concerning China and Turkey, there had been a change from ID in the population between 2006 and 2008 [27, 28] to adequate iodine between 2015 and 2018 [14, 19]. In China, the salt iodisation programme had become sustainable for the entire population by 20 years after intervention. It began in 1990 and the iodine concentration has been adjusted seven times during the intervention [16, 18–20]. This readjustment started with 40–60 mg iodine/kg salt when the country had ID [28] and was then adjusted to 15–40 mg iodine/kg salt in 2010. The programme has achieved sustainable IDD elimination in 32 provinces. From 2011 to 2018, the ICS was readjusted again by reducing iodine concentration in salt, and adequate iodine was achieved again in all provinces in 2014. In 2017, it was effective for pregnant women and, in 2018, sustainable for the iodine concentration in areas near water. However, stopping salt iodisation programmes in areas with water that has a high iodine concentration can be a big problem. However, stopping salt iodisation programmes in areas with higher iodine concentration in water and soil may not be the solution, but if in these areas readjusted iodine in all sources including salt may correct iodine, it has bad consequences. These findings show one experience of the good progress of the salt iodisation programme based on reduced iodine in salt as part of the USI strategy. This evidence is supported by other authors; for example, in Morocco in 1995, the authorities recommended a level of iodisation of the 80 ppm when the level of TGR in the country was higher, in 2009, this was reduced to 15–40 ppm, and now, the country people have adequacy, other countries are with Qatar since 1996, the country has adequate UIC in population, and now the country pulls out all the stops to reduce iodine concentration in salt. United Arab Emirates, Kuwait, Oman, Bahrain, and Palestine archived adequacy UIC in people since 1996, and they have the initiative to reduce iodine in salt [42].

In Turkey, the salt iodisation programme became sustainable for the population 14 years after the intervention began in the urban areas in the south, based on increased adequate iodised salt coverage. Since 2001, it has never been readjusted [14]. In 2001, urban areas in the west, with 20–40 mg iodine/kg salt, had ID and IDDs, and the iodised SC was >68% [27]. After 4 years with the same iodine concentration in salt, in urban areas in the south, the iodised salt coverage was >90% and the programme was sustainable.

The salt iodisation programmes in Turkey and China have shown remarkable success. Although the countries have used different strategies—China has reduced the ICS over time whilst Turkey has maintained the ICS—in both countries, the ICS was adequate [42, 43].

Of note, IDDs also occur in countries with higher consumption of other food sources of iodine, such as milk, dairy products, and water. It is necessary to readjust the salt iodisation polices that may be underlying these aspects.

The monitory of UIC and SIC is recommended does three and three years, but in every ten years of the implemented salt iodisation programmes, the UICs in the population are adequate (median UIC based on scholar children), but the people with excess iodine intake increased, or TGR is >5% because it occurs also in the situation of iodine excess intake, or because of their consequences with thyroid dysfunction in population or increased iodine deficiency in some groups with pregnant women and lactating women. Salt iodisation is necessarily readjusted and assesses other contributed factors of iodine variation in diet.

### 4.6. Limitations

The limitations of this review are the limited number of representative studies; few studies for the Americas and Europe and different methodologies were used to calibrate the iodine content in salt, resulting in different iodine quantifications. However, five of the 13 countries had been assessed more than one time, and some in different regions of the same country. According to UNICEF recommendations, the inclusion of many scenarios to assess ID in people is necessary.

## 5. Conclusions

This review shows that UIC levels have increased from 16.9% to 166% and also TGR has reduced by 1.2% to 62.3% within 18 years in populations with ID. Hence, salt iodisation programmes have had good impacts on health. The outcomes confirm that 18 years after the implementation of salt iodisation programmes in the 13 countries examined in the included reviews, most had achieved sustainable elimination of IDDs with an adequate UIC. Nevertheless, more detailed studies are still needed to confirm that all communities are equally protected against IDDs. Therefore, over time monitoring of impact indicators is relevant. Eliminating IDDs throughout the entire world is the next stage. For this endeavour, the iodine concentration in salt must be adequate according to legislations and coverage iodised salt increased in the household, as well as monitoring all sources of iodine intake in the diet is required to provide a better prediction of the iodine deficiency whilst avoiding excess iodine intake. However, iodised salt still remains the first source of iodine in diet able to be adequate in iodine and reduces TGR. In addition, pregnant and lactating women must be especially considered because they are in great need of iodine by susceptibility to iodine losses.

## Figures and Tables

**Table 1:** Main characteristics, results, and conclusions of the studies that used salt iodisation programmes.

Location of studies and references	Study design/period of study	Main results			
Isparta- western of Turkey (rual/urban areas)- [27]	“To determine TGR and UIC” in 500 children from six to 11 years old 2001	**SC**: 68% more in urban areas (*P* < 0.05) compared to in rural areas where it was not refined	**MUIC**, 70 *µ*g\L: ↑42 *µ*g\L (1997–2001). - m il IDD	**TGR**: 26% (ultrasonography) and 30.4% (palpation): ↑1 and 4.6% (1997–2001) TGR:7.6% in iodised salt consumers and 33% in no consumers. Moderate IDD	Salt iodisation programme without success

Panshan, Zhangwu, and Huanghua- were north, southwest, and coastal region in China (rural areas)- [28]	“To assess the effect of iodine intake, goitre, and thyroid nodules” in adults and children (eight to nine years old) 3338–1999 2708–2004	**In** Panshan, Huanghua, and Zhangwu: **ICS**, had a median of <3.4, 25, and 45.6 mg/kg: ↓1.7 and 8.9 mg\kg (Huanghua and Zhangwu) from 1999 to 2004. **The iodine content in the water** was 10, 7.8, and 201 *μ*g/L	**In** Panshan, Huanghua, and Zhangwu: **MUIC**, 87.6, 633.5, and 213.9 *µ*g\L in children. And 96.7, 635.2, and 350.3 *µ*g\L in adults	**In** Panshan, Huanghua, and Zhangwu: **TGP**: 20%, 8.3%, and 10% in children. Moderate IDD in iodine deficiency areas. Mild IDD in iodine excessive areas	Salt iodisation programme without success in iodine deficiency areas but was inequitable in excess areas

Tanzania (rural/urban areas) [10]	“To assess the use of iodised salt, UIC and TGR” In 140758 children and adolescents (six to 18 years old). 2004	**ICS**: had a mean do not show but it was 84%.**SC**, 84%	**MUIC** 203.6 (192–215) *µ*g\L	**TGP**: 12.3%: ↓48.4 % (1980–2004). -Mild IDD	Salt iodisation programme was effective

North of India (rural/urban areas) [26]	“To assess IDD's control program” in 2860 adults and children (one to 40 years old). 2007	**ICS** was 6.3 mg/Kg (30%) **SC**, 25% adequate consumption iodised salt	**MUIC** 100 (5.0–560) *µ*g/L	**TGR**: 27.7%: ↓41.3% (1982–2006). **TGR**, 31% in children with UIC<100 *µ*g\L -Moderate IDD	Salt iodisation programme was inequitable

Raipur- were Central region in the India (urban/rural areas)- [29]	“Estimate TGR and UIC. ICS. Awareness about IDDs.” in 1177 pregnant women 2006–2007	**ICS**, was >15 mg\kg of iodine (68%) **SC**, 66% consumption adequate iodised salt, majority shopkeepers were not aware about iodised salt and legislation regarding it	**MUIC**, 94 *µ*g\L	**TGR**: 0.17%	Salt iodisation programme without success

New Zealand (urban/rural areas) [13]	“To assess of mandatory fortification of bread” in 147 children (eight to ten years old) 2010–2011	**ICS**, >45 mg\kg **(90%) SC**:28% and associated UIC (*P*=0.017)	**MUIC**, 113.6 (78–159) *µ*g\L: ↑45 *µ*g\L (2002–2011)		Salt iodisation programme was effective

Mexico (urban areas) [23]	“To describe NS in iodine and relate NS “in 1544 children (six to 12 years old) 2010–2011	ICS: was 28.4 ± 9.4 (77.8%) high iodine consumption was the real problem	MUIC, 297 (5–1519) *µ*g\L. Excessive and deficiency iodine coexist		Salt iodisation programme was effective

Zhejiang- eastern in the China (urban/rural areas)- [30]	“To assess the NS in iodine after the 3rd readjustment” in children (from eight to ten years old) 420–2011 1560–2013	ICS was 24 mg/kg: ↓6.4 mg\kg	MUIC, 174.3 (range 586) *µ*g\L: ↓62.8 *µ*g\L from 2011 to 2013. UIC was more in urban areas compared to rural		Salt iodisation programme was effective

South of Iran (urban areas) [31]	“To assess the NS of iodine two decades after implemented” in such 100 pregnant women, lactating women, and their newborns with three to five days of age. 2000–2015	**ICS**: a Median of 26 (21–30) mg/kg and 25 (18–28) in pregnant women and lactating women. **ICS** (*r* = 0.24, *P*=0.019) UIC of newborns was associated with ICS of theirs mother (*r* = 0.49, *P*=0.001)	**MUIC**, 103 (59–55) *μ*g/L and 77 (42–194) *μ*g/L in pregnant women and lactating women was below compared newborns 198 (84–260) *μ*g/L		Salt iodisation programme without success

Tunisia (rural/urban areas) [22]	“To obtain WHO's sustainable elimination IDD “in1560 children (six to 12 years old). 2012	**ICS** was 22 mg/kg (44.1%) **SC**, in regions with inadequately iodised salt represented 30% and excessive 66,3%	**MUIC**, 220 *µ*g\L (199–241 *µ*g\L)		Salt iodisation programme was effective

Sierra Leone (urban/rural areas) [24]	“To assess NS in iodine” in 571 adolescents, 220 nursing, and 154 pregnant women 2013	**ICS** was >15 mg\kg of salt (81%)	**MUIC**: - 180.6 *µ*g\L– Pregnant women; - 217.2 *µ*g\L –Teenage girls and; - 196.8 *µ*g\L – Nurses mothers		Salt iodisation programme was effective

32provinces-China (urban/rural areas) [18]	“Analysis achieved challenge IDDs “in 31 million pregnant women and children 2005–2010	**SC**, >90%	**MUIC**, 239 *µ*g\L:↓6 *µ*g\L (2005–2010) in children and 184 *µ*g\L in pregnant women	**TGR**: <5%. Effective elimination IDDs in schoolchildren	Salt iodisation programme was sustaining

Portugal (urban areas) [21]	“To assess NS in iodine and the use of iodised salt” in school-age children (six to 12 years old). 2015–2016	**ICS** was 16–54 mg/kg in 2% of people **SC**, 16% and was 50% in milk products consumer	**MUIC** 129 (80–180) *µ*g\L: ↑23 *µ*g\L (2012–2016)		Salt iodisation programme was effective

North-west of Iran (rural/urban areas) [17]	“To assess the TGR and UIC” in 240 children of eight to ten years old. 2007–2015	**ICS:** had a mean of 27 mg/kg (97%). **SC**: 98% of adequately iodised salt	**MUIC**, 145 *μ*g/L: ↑5 *μ*g/L from 2007 to 2015, ↑6.2% from 2007 in rural compared to 25.7% in urban. UIC>300 *μ*g/L: ↑22.1% from 2007 in urban: And 5.7% in rural. Iodine deficiency is rural and excess in urban areas	**TGR**: 0.4%: ↓43,6% from 1996	Salt iodisation programme was sustaining

Cassino-Italy (urban areas) [32]	“To assess the NS of/iodine after 10 years programme deployed” in 96 pregnant women cases and 69 nonpregnant in control. 2016–2017	Those who had consumed iodised salt with milk had more UIC (*P* < 0.05). **SC**: 42% cover (<90% adequate ICS)	**MUIC**, 110.6 (15.8–491.3) *μ*g/L was more compared to 97.7 (28.1–1154.3) *μ*g/L in control. -Insufficient UIC	**TGR**: Pregnant women had more thyroid volume. -Mild IDD	Salt iodisation programme without success

Kebele village Ethiopia(rural areas) [33]	“To assess the TGR and associated factors” in 356 pregnant women 2014	**ICS**: a median of 12.2 (6.9–23.8) *μ*g/L (39.3%) **SC**: 94% used crystal salt and kept well. Iodised salt cooking addition, light stored, ICS below 15 mg/kg, illiteracy, and age >30, associated with iodine intake	**MUIC**, 85.7 (45.7–136) *μ*g/L: ↓ 94 *μ*g/L the 1^st^ (168) to 3rd (74) trimester - insufficient UIC	**TGR**: Median of 20 (16–24) %: ↑9% the 1st (13) to 3rd (22) trimester -moderate IDD	Salt iodisation programme without success

31 provinces- Iran (rual/urban areas) [15]	“To assess NS of iodine” in 18.000 children (eight to ten years old) 2007–2014	**ICS:** had a mean of 30 mg/kg (63%) **SC:** 98% used iodised salt and 82% were crystal	**MUIC**, 161 *μ*g/L in 2013: ↑ 79 *μ*g/L from 2013 (all provinces had UIC > 100 *μ*g/L)	**TGR**: 5,7%: ↓ 62.3% (1989–2007) in 2013 not have a GTR reporter	Salt iodisation programme was sustaining

31 provinces - China (urban) [16]	“To evaluate the impact of the ICS and NS of iodine “in children (eight to ten years old): 2011 : 14.950 2014 : 48.975 and pregnant women 2011 : 13.932 2014 : 19.500	**ICS**: had a mean of 25 mg/kg: ↓5.4 mg/kg from the 2011 to 2014 (>90%) **SC:** > 90% used adequate iodised salt. The policy was based on reduction ICS	**MUIC**, 197.9 *μ*g/L: ↓40.7 from 2011 to 2014 in children and 154.6 *μ*g/L in pregnant women: ↓29.8 *μ*g/L from 2011 to 2014	**TGR**: 2.6% in children the same in 2011	Salt iodisation programme was sustaining

Amhara region - Ethiopia (rural areas) [11]	“To assess the TGR and UIC” in infants of six to 11 months old in case (*n* = 743) and control (*n* = 654) 2010–2013	**ICS**: mean does not show. **SC** > 90% (in the intervention group). Observed improve UIC level in children the mother that receive iodine salt	**MUIC**, 228.0 (123.4–411.5) *μ*g/L: ↑102 *μ*g/L from baseline in intervention group more than 155.0 (81.4–283.9)*μ*g/L: ↑140.2 *μ*g/L in control (*P*=0.001)	**TGR**: <5% in both groups	Salt iodisation programme was sustaining

Cameroon (rural areas) [25]	“To monitor the NS of iodine “in children of six to 12 years old. 1990–2018	**ICS**: had a mean of 72 mg/kg:↑153% (1999–2017). The imported salt contribute to more than 200% of the salt	**MUIC**, 1145 *μ*g/L: ↑166% from 1999 to 2017. Stem from 2006 to 2018 UIC>300 *μ*g/L: ↑ 756 *μ*g/L. From 2006 the people presented with risk of an adverse effect of health		Salt iodisation programme was inequitable

Shanghai– Central coast in the China (urban areas) Central coast [20]	“To assess NS of iodine, summarized “in children eight to ten years old, the number of years: 1999 : 1320 2005 : 1223 2011 : 1234 2014 : 1510 2017 : 1215	**ICS**: had a mean of 24.3 ± 5.6 mg/kg: ↓19,5 mg/kg from 1999 to 2017 **SC**: >90% (1999–2011) and <90% in 2014–2017 observed reduction of 19,5 mg of iodine/kg in over time	**MUIC**, 183.0 (114–267) *μ*g/L: ↓53 *μ*g/L in the high iodised salt group, ↑25 *μ*g/L in the low iodised salt group, and ↑16 *μ*g/L in the noniodised salt group from 1999 (*X*^2^ = 35.4: *P*=0.000) to 2017 (*X*^2^ = 10,5: *P*=0.005)	**TGR**: 1.9%: ↓ 1,2% from 1999 to 2017	Salt iodisation programme was sustaining

Tianjin- northeastern in the China (urban areas) - [19]	“To assess the NS of iodine to stopped iodised programme in high water iodine areas” in 399 children aged seven–12 years 1990–2018	**ICS:** had a mean do not show **areas WIC** SC(%) total II(*μ*g/d) **<** **100 *μ*g/L** 56.6 441.1 **100-150 *μ*g/L** 21.4 369.4 **150-300 *μ*g/L** 11.8 473.9 **≥** **300 *μ*g/L** 5.1 576.8. The salt iodisation programme stopped in areas with iodine water ≥300 *μ*g/L, in areas with iodine in water <100 *μ*g/L had also higher consumption of iodine but eliminated sustaining IDD	**Areas WIC** UIC (*μ*g/L) < 100 *μ*g/L 217.2 (157.93–290.58 100–150 *μ*g/L 187.65 (133.20–237.05) 150–300 *μ*g/L 209.15 (137.23–258.38) ≥ 300 *μ*g/L **476.30 (332.20–639.30)**	**TGR: in areas with UIC** ≥ 300 *μ*g/L was 10% and 27% thyroid nodules (*P* < 0.05). In areas with WIC ≥300, 150–300, 100–150, and < 100 *μ*g/L were 11%, 12%, 38%, and 97% respectively. Observed mild IDD in people had risk of adverse effect of health	Salt iodisation programme was without success in areas with iodine concentration in water ≥300 *μ*g/L but in areas with <100 *μ*g/L it was sustaining

Antalya- southern in Turkey (urban) [14]	“To assess the NS of iodine “in 1.594 children of six to 14 years old. 1999–2015	**SC**: >90%.	**MUIC**, 163.3 (105.3–254.8) *μ*g/L: ↑ 101 *μ*g/L from 1999 to 2015 (*P*=0.0001). In 2015 not registered iodine deficiency	**TGR**: 0,3%: ↓33,7% from 1999 to 2015 (*P*=0.0001)	Salt iodisation programme was sustaining

ID: iodine deficiency; IDD: iodine deficiency disorders; NS: nutrition status. ↓: low; ↑: raised; <: less; >: higher; ±: standard deviation; ICS: iodine concentration in salt; MUIC: median Urinary iodine concentration; UIC: urinary iodine concentration; WIC: water iodine concentration; TGP: total goitre rate; SC: salt coverage or consumption, II: iodine intake.

## Data Availability

No data were used to support this study.

## Supplementary Materials

Supplementary Materials Figure 1 represents the correlation of the Urinary Iodine Concentration (UIC) with years of the implementation of salt iodisation programmes, where it showed an increase of UIC in the population as time goes on. Figure 2 is the opposite; it represents a decrease in the correlation of total goitre rate (GTR) with years of the implementation of salt iodisation programmes.
