# Supplementary material for: Assessment of the Impact of Salt Iodisation Programmes on Urinary Iodine Concentrations and Goitre Rates: A Systematic Review
Source: J Nutr Metab. 2021 Jun 2;2021:9971092. doi: 10.1155/2021/9971092 (PMC8192202; doi:10.1155/2021/9971092)
Supplement: Supplementary Materials — Figure 1 represents the correlation of the Urinary Iodine Concentration (UIC) with years of the implementation of salt iodisation programmes, where it showed an increase of UIC in the population as time goes on. Figure 2 is the opposite; it represents a decrease in the correlation of total goitre rate (GTR) with years of the implementation of salt iodisation programmes. [file 9971092.f1.docx]

Supplemental Figure 2. TGR evolutions in years of the salt iodisation programmes in the world

Supplemental figure 1. UIC evolutions in years of the salt iodisation programmes in the world
